# The effects of repeated brain MRI on chromosomal damage

**DOI:** 10.1186/s41747-022-00264-2

**Published:** 2022-03-03

**Authors:** Cecile Herate, Patricia Brochard, Florent De Vathaire, Michelle Ricoul, Bernadette Martins, Laurence Laurier, Jean-Robert Deverre, Bertrand Thirion, Lucie Hertz-Pannier, Laure Sabatier

**Affiliations:** 1 PROCyTox, DRF, French Alternative Energies and Atomic Energy Commission (CEA), Paris-Saclay University, Fontenay-aux-Roses, France; 2 National Institute for Health and Medical Research, Center for Research in Epidemiology and Population Health (CESP), INSERM U1018, Radiation Epidemiology Teams, Villejuif, France; 3 Institute Gustave Roussy, Villejuif, France; 4 University Paris Saclay, Villejuif, France; 5 CEA/DRF/IJ/Neurospin/UNIACT, and UMR1141, Inserm, Paris University, Gif-sur-Yvette, France; 6 CEA/DRF/DIREI Research Infrastructures Europe and International Fundamental Research Division, French Alternative Energies and Atomic Energy Commission (CEA), Paris-Saclay University, Gif sur Yvette Cedex, France

**Keywords:** Centromere, Cytogenetic analysis, Chromosome aberrations, Magnetic resonance imaging, Telomere

## Abstract

**Background:**

Magnetic resonance imaging (MRI) is currently considered a safe imaging technique because, unlike computed tomography, MRI does not expose patients to ionising radiation. However, conflicting literature reports possible genotoxic effects of MRI. We herein examine the chromosomal effects of repeated MRI scans by performing a longitudinal follow-up of chromosomal integrity in volunteers.

**Methods:**

This ethically approved study was performed on 13 healthy volunteers (mean age 33 years) exposed to up to 26 3-T MRI sessions. The characterisation of chromosome damage in peripheral blood lymphocytes was performed using the gold-standard biodosimetry technique augmented with telomere and centromere staining.

**Results:**

Cytogenetic analysis showed no detectable effect after a single MRI scan. However, repeated MRI sessions (from 10 to 20 scans) were associated with a small but significant increase in chromosomal breaks with the accumulation of cells with chromosomal terminal deletions with a coefficient of 9.5% (95% confidence interval 6.5–12.5%) per MRI (*p* < 0.001). Additional exposure did not result in any further increase. This plateauing of damage suggests lymphocyte turnover. Additionally, there was no significant induction of dicentric chromosomes, in contrast to what is observed following exposure to ionising radiation.

**Conclusions:**

Our study showed that MRI can affect chromosomal integrity. However, the amount of damage per cell might be so low that no chromosomal rearrangement by fusion of two deoxyribonucleic breaks is induced, unlike that seen after exposure to computed tomography. This study confirms that MRI is a safe imaging technique.

**Supplementary Information:**

The online version contains supplementary material available at 10.1186/s41747-022-00264-2.

## Key points

- A longitudinal follow-up of genotoxicity was conducted on volunteers receiving repetitive (up to 26) 3-T magnetic resonance imaging (MRI) brain scans.
- One single MRI session of 90 min had no impact on chromosomal integrity.
- Repetitive MRI scans (*n* = 20) over a 2-year period showed a small increase in chromosome breaks that reached a plateau thereafter. These breaks concerned only chromosome terminal deletions but not dicentrics (which can be observed after computed tomography exposure) and are considered hallmarks of irradiation damage.
- MRI remains one of the safest imaging techniques.

## Background

Magnetic resonance imaging (MRI) is currently used on a routine basis in Europe and across the world and the use of MRI scans is increasingly applied in both clinical and preclinical studies for diagnostic purposes. Few studies have explored the genotoxic effects of MRI, either *in vivo* or *in vitro*, in which the focus has included deoxyribonucleic acid (DNA) damage markers and chromosomal aberrations (CAs). Results concerning the genotoxic potential of MRI scans are inconsistent.

Among the studies evaluating single-strand breaks (SSBs) [1–3], only Lee et al. [1] detected an increase in SSBs following a single *in vitro* 3-T MRI exposure. Several studies failed to detect any significant increase in γ-H2AX, a surrogate marker for double-strand breaks (DSB), either after *in vitro* or *in vivo* exposure [4–7]. However, Lancelotti et al., among others, reported a temporary increase in the level of γ-H2AX 2 days to 1 month after *in vivo* exposure [8, 9]. Concerning CAs, an increase in the number of Micronuclei (MN) was reported in two of the three studies in which it was tested *in vitro* and *in vivo* after a single 3-T or even at a lower 1.5-T MRI session, respectively [1, 10]. Lee et al. [1] additionally followed unstable CAs using Giemsa staining after a single 3-T MRI exposure *in vitro* and reported a significant increase in the frequency of chromatid breaks.

In the context of such conflicting results, we took the unique opportunity to organise an ancillary and longitudinal study of young adults exposed to repetitive brain 3-T MRI scans. We searched for long-term chromosomal changes using the reliable technique of peptide nucleic acid (PNA) fluorescence *in situ* hybridisation (FISH) staining of telomeres and centromeres to improve the detection sensitivity of CAs.

## Methods

### Cohort selection

The objective of the IBC project (Individual Brain Charting, part of the Human Brain Project, https://www.humanbrainproject.eu/) is to carry out a nearly exhaustive functional brain mapping, by performing 50 MRIs over 5 years on 12 healthy adult volunteers (*i.e.*, without cognitive or neurological/psychiatric condition and without drug abuse), aged 27 to 40 years (mean age 33 years) at the start of the study (Table 1). The IBC project received ethical approval IDF VII N°14-03 and ANSM IDRCB 2014-A00563-44 and each volunteer provided written consent (for a detailed project description, see Pinho et al., 2018 [11]). The group was informed yearly of the study progress and scant attrition was noted. Of the 15 volunteers initially enrolled, two withdrew prior to the first MRI, and one after ten MRIs. One subject (S8) underwent eight conventional whole body computed tomography (CT) scans (with and without iodined contrast injection) for a pelvic surgery with an approximate effective radiation dose of 20 mSv for each scan. The mid-term results of the ancillary study dedicated to the follow-up of possible genotoxic effects of MRI are presented for 26 MRI scans as Supplementary table.

**Table 1:** Description of the cohort of volunteers

Subject number	Sex	Age at the 1st MRI (years)	Months between 1st and 10th MRI	Months between 1st and 20th MRI	Months between 1st and 25th MRI
S1	M	27	13	33	36
S2	M	28	10	25	33
S3	M	38	11	28	35
S4	M	33	13	31	39
S5	F	38	11	28	39
S6	M	28	10	33	38
S7	M	39	21	34	48
**S8**	**M**	**36**	**7**	**22**	**34**
S9	M	30	7	18	23
S10	M	27	11	36	44
S11	M	41	7	23	32
*S12*	*M*	*32*	*25*	*–*	*–*
*S13*	*F*	*36*	*14*	*–*	*–*

Thirteen participants were selected for this study, which lasted 3 to 4 years, depending on the participant. They are all non-smokers and young adults. Ten were included for the entire study, as they received 26 MRI exams but no radiation exposure. Subject number 8 (reported in bold characters) was exposed to repetitive computed tomography exams and was excluded from the global analysis. The two volunteers (number 12 and number 13; in italics) have not yet finished the study and were considered only for the analysis of the effects following the first MRI session. *MRI* Magnetic resonance imaging

### MRI sessions

Volunteers underwent MRI on a 3-T MRI machine (Trio Siemens, Erlangen, Germany), with a 32-channel head coil, at the NeuroSpin Research Center of the CEA-Saclay, France. This scanner is similar to those currently in use in clinics. The use of multi-band sequences and/or acceleration techniques allowed a resolution of 1.5 mm isotropic for an acquisition time of approximately 2 s per volume (total scan time 90 min). All subjects underwent the same MRI acquisitions, but order and time interval varied due to personal availability and scanning constraints.

In the first session, imaging acquisition was dedicated to brain anatomy with three-dimensional (3D) T1-weighted MPRAGE acquisition (1 mm^3^ isotropic, repetition time 2,300 ms, echo time 2.8 ms, flip angle 9°, matrix 256 × 256 × 176), 3D T2-weighted acquisition (repetition time 3,200 ms, echo time 419 ms, matrix 230 × 230 × 160, 0.9 mm^3^ isotropic, parallel imaging acceleration factor 2), and 3D fluid-attenuated inversion recovery acquisition (repetition time 5,000 ms, echo time 396 ms, matrix 230 × 230 × 160, 0.9 mm^3^ isotropic, parallel imaging acceleration factor 3). Standard diffusion-weighted images were also acquired for screening (*b* = 1,500 s/mm^2^, 20 directions, 2 mm^3^ isotropic, matrix 240 × 240 × 140, repetition time 9,000 ms, echo time = 66 ms, parallel imaging acceleration factor 2). Nearly all subsequent sessions were dedicated to multitask axial functional MRI acquisitions with high spatial resolution, with a typical sequence protocol as follows: gradient-echo echo-planar imaging, repetition time 2,000 ms (or less), echo time 27 ms, flip angle 74°, 1.5 mm^3^ isotropic, parallel imaging acceleration factor 2, MB = 3. To comply with the aim of the IBC project of building a high-density, individual anatomo-functional atlas of unprecedented precision, more than 200 cognitive functional contrasts were accumulated over the 90-min acquisition sessions. In each session, acquisitions typically combined a 3D T1-weighted sequence and repeated functional gradient-echo echo-planar imaging sequences. Sequences are additionally detailed in Pinho et al., 2018 [11].

### Blood collection and culture

Peripheral blood samples were obtained at baseline (three samples each, 2 weeks apart, with each volunteer serving as his own control). Subsequent samples were obtained prior to the following MRI session and/or immediately after the session. The samples, collected in heparin-lithium tubes, were processed by the company Biomnis (Ivry-sur-Seine, France) in accordance with our laboratory protocol and cultured (in duplicate) for 48 h with bromodeoxyuridine and phytohemagglutinin. Metaphases were prepared using standard procedures [12]. After fixation, the cells were spread on five slides and stored at -20 °C until shipment to our cytogenetic laboratory for staining, microscopic image acquisition, and analysis. The remaining fixed cells were kept frozen at -20 °C for biobanking.

### Dicentric assay staining and acquisition

In the current protocol, the detection of chromosome aberrations was improved upon compared to the Giemsa technique by labeling the telomeres and centromeres [13]. They were stained using the FISH technique with a Cy-3-labeled PNA probe specific for TTAGGG for telomeres and a fluorescein isothiocyanate (FITC)-labeled PNA probe specific for centromere sequences (Centro-FITC: FITC-AAACTAGACAGAAGCAT) (both from Panagene, Daejon, South Korea), as described previously [13]. Images of the telomere/centromere (TC)-stained metaphases were captured using the automated acquisition module Autocapt software (MetaSystems, version 3.12.7, Heidelberg, Germany) and analysed with an IMAGEJ lab-developed plugin, CEA-Detector.

Dicentric assay and telomere and centromere staining are described step by step in: https://www.youtube.com/watch?v=ZG5ssFNI3Jc and https://www.youtube.com/watch?v=RqI1ulPWD_E.

### Metaphasis analysis

Only metaphases with 44 to 46 chromosomes were scored on a minimum of two slides, one per culture. TC staining allows scoring of various unstable aberrations: dicentrics, rings, and different types of acentric fragments (Fig. S1). Acentric fragments are chromosomal fragments without a centromere. They can possess two telomeres at each extremity, resulting from the fusion of two chromosomal fragments coming from two DSBs. Acentric fragments with one telomere result from one DSB caused by terminal deletion of a chromosome, whereas acentric fragments without any telomere result from two DSBs in the same chromosomal arm. Only the acentric fragments in excess, *i.e*., those that did not result from dicentric (Dic) or centric rings formation, are included in the final scoring (acentric fragments in excess = type E acentric). As metaphase chromosomes are analysed, chromatids are duplicated and one telomere gives two telomere signals, one on each chromatid, as shown in Fig. S1. These CAs are all considered to be unstable with a decrease at each cell division, the most documented being Dics with a 50% loss during each cell division). Considering that the percentage of metaphases in first or second generations can be subject to large variations (variability of the proliferation rate linked to the different batches of culture medium used over the years and to inter-individual variations*)*, cells were only analysed in the first generation (identified by bromodeoxyuridine incorporation). Scoring the various unstable aberrations allows the calculation of the total DSBs per cell. Approximately 1,000 metaphases were analysed for each time point (Table S1). Analysing the entire study, we also scored 10 “rogue cells”, *i.e.*, multi-aberrant cells [14]. These cells were not considered for graphs, as they are not considered to be radiation-induced, which could happen after heavy ion irradiation, but which was not the case here. As such, we performed parallel statistical analysis either including or removing these data, and the results and conclusions remained similar.

### Chromosome painting

The chromosome painting technique is used to identify individual chromosomes and allows scoring of both unstable (dicentric) and stable (translocations) labeled chromosomes. Staining was performed using MetaSystems chromosome painting probes for chromosomes 1 (TexasRed, TR), 4 (mix of TR and FITC probes), and 11 (FITC) on the same slides used for TC staining, following the manufacturer’s suggested protocol.

### Statistical analysis

Statistical analysis was performed using generalised linear mixed models [15] for repeated measures, the number of DNA events per metaphase being assumed to follow a binomial distribution. Hierarchical analysis was performed to account for intra-individual and intra-sample correlations. This methodology allowed modeling of the ratio between the number of events and the number of metaphases studied per slide, considering the fact that the slides are part of samples, which themselves belong to the same individual. All analyses were adjusted for sex and age at the time of sampling. The reported associations between MRI and the frequency of various cytogenetic parameters were estimated assuming linear dose-response. This option was chosen as the small number of volunteers did not permit investigation into the shape of the dose-response and log-linear assumption tests provided similar results. All analyses were performed with or without rogue cells (multi-aberrant cells) and with or without volunteer S8, to test robustness. The possibility for outliers skewing results was eliminated by performing sensitivity analysis and iterating analyses excluding each volunteer, in turn.

## Results

### Cohort

At the time of this publication, 11 volunteers have completed the 20 first MRI sessions, including S8, who was analysed separately due to the performed CT scans for an unrelated non-brain related condition (Table 1). Two volunteers (number 12 and number 13) have not yet completed the 20 MRIs. While only the 10 volunteers who underwent 20 MRI sessions were included in the complete study, all 13 were considered for the study of the short-term changes associated with a single MRI. Data, including the number of metaphases scored, as well as the number of damaged cells for each time point and subject, are presented in Table S1 and the sampling table is shown in Fig. S2.

### No chromosomal change after a single MRI scan

**Fig. 1:**
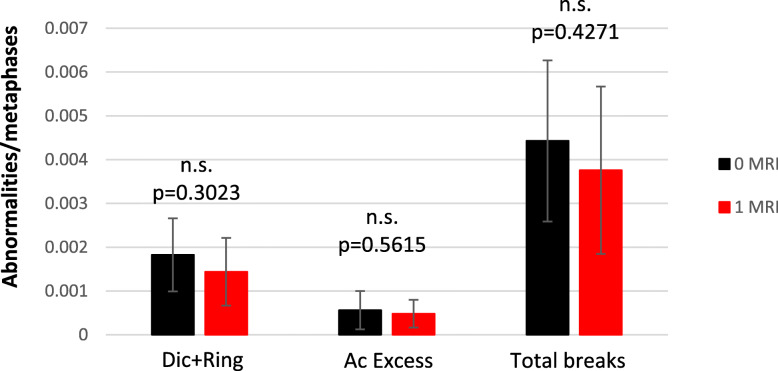
One MRI session does not trigger any genotoxic effect. The dicentric chromosome assay was performed on blood cells of volunteers sampled 30, 15, and 0 days before the exam to take into account the heterogeneity of the background (mean in black). The potential effect of one MRI session was assessed the day of the first MRI and prior to the second MRI (mean in red). Unstable aberrations (Dic + Tric + ring) were scored after telomere and centromere staining, as well as acentric fragments (type E acentrics, acentrics in excess), those resulting from Dics or ring being excluded). Total breaks were calculated from these data. *Ac*, Acentric fragment; *Dic*, Dicentric chromosome; *MRI*, Magnetic resonance imaging; *n.s.*, No significant differences; *Tric*, Tricentric chromosome

Accounting for intra-individual variations after a single 3 T brain MRI of 90 min, there was no significant increase in the frequency of damaged cells (cells with at least one chromosome aberration), with a similar frequency of dicentric (1.7/1,000) and acentric fragments (0.5/1,000) in all samples. The frequency of chromosomal breaks remained low and constant (Fig. 1), with an initial average of approximately 4 breaks for 1,000 metaphases, corresponding to previously reported background frequency [13, 16, 17].

### Repeated MRI exposure was associated with slight chromosomal instability

Focus is initially on the first 20 MRI scans, as a lapsed period of 2 years can be considered as the time necessary for the turnover of lymphocytes. Mid-term data with the additional MRI exams 25 and 26 will be discussed subsequently.

#### Increased chromosomal instability characterised by terminal deletions

**Fig. 2:**
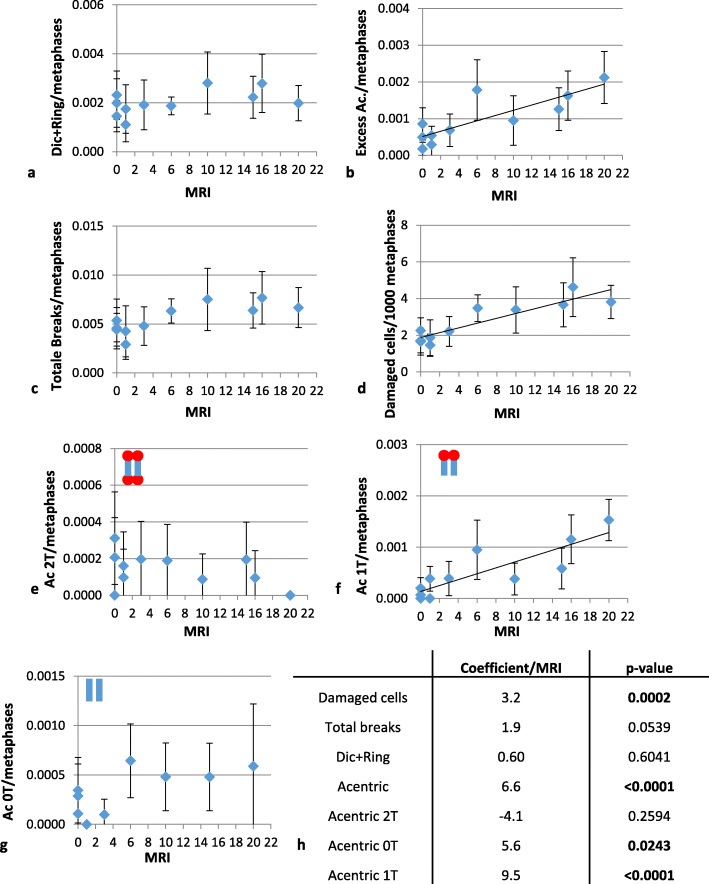
Increasing chromosomal instability due to terminal deletions during the first 20 MRI sessions. Between 846 and 3,357 metaphases were observed for each time point and participant after telomere/centromere staining. Various cytogenetic parameters were scored: dicentric chromosomes and rings (**a**); total type E acentrics (excess acentric fragments) (**b**), total chromosomal breaks (**c**), damaged cells (**d**), 2-T acentric fragments resulting from two chromosomal breaks and fusion (**e**), 1-T acentric fragments equivalent to a terminal deletion (**f**), and 0-T acentric fragments resulting from two chromosomal breaks in the same chromosomal arm (**g**). **h** Table presenting the stratified statistical analysis (correlation coefficients and *p* values). *Ac*, Acentric fragment; *Dic*, Dicentric chromosomes; *MRI*, Magnetic resonance imaging; *T*, Telomere

We detected no significant correlation between the first 20 MRIs and the Dics plus ring frequency, which remained stable (*p* = 0.604 *F* test) (Fig. 2). The lack of an increase in the frequency of fused chromosome (*p* = 0.341, *χ*^2^ test) was confirmed by analysing translocations by chromosome painting of chromosomes 1, 4, and 11 for two volunteers selected at random before any MRI *versus* after 16 MRI scans (Fig. S3). However, repeating MRI sessions was associated with a significant increase in acentric fragments (Fig. 2b). The frequency of total acentric fragments increased, on average, by 6.6% (95% confidence interval [CI] 4.3–8.9%) per MRI (*p* < 0.001) (Fig. 2h), reaching a more than threefold increase in the frequency of type E acentric fragments after 20 MRI exams. The total DNA breaks per metaphase (Fig. 2c) did not significantly change after 20 MRI sessions but showed a trend towards increasing (Fig. 2h, *p* = 0.054, *F* test). The frequency of acentric fragments with two telomeres remained stable (*p* = 0.310), whereas those without telomeres increased slightly after the repeated MRI sessions (5.6%, 95% CI 0.7–10.5%, per MRI, *p* = 0.016) both being due to two DSBs (Fig. 2e–g). The formation of acentric fragments with one telomere (1 DSB) showed the highest correlation with MRI (Fig. 2f–h). This increase was highly significant, with a coefficient of 9.5% (95%CI 6.5–12.5%) per MRI (*p* < 0.001). The increase in the frequency of terminal deletions was easily detectable in each volunteer (Table S1).

#### Increased frequency of damaged cells

**Fig. 3:**
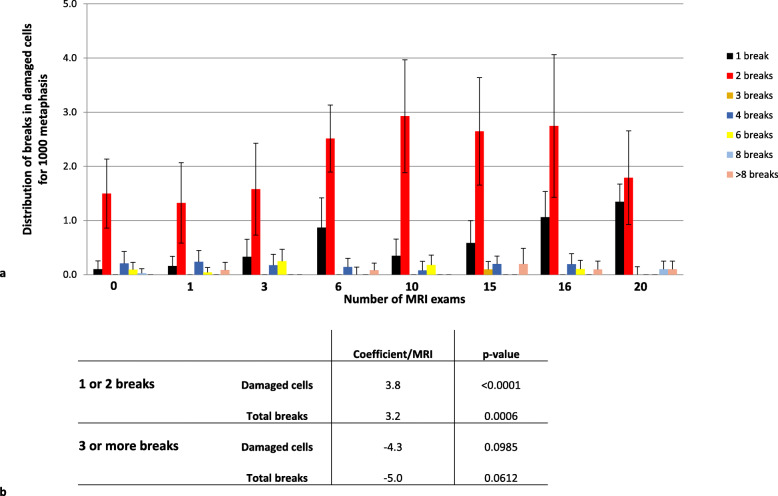
Distribution of deoxyribonucleic acid breaks in damaged cells. **a** Only the data for damaged cells are shown and are clustered according to the number of breaks scored. In this graph, all damaged cells are considered, including rogue cells. **b** Statistical analysis of the damaged-cell phenotype. Cells with one or two breaks were separated from those with three or more breaks. The correlation between the number of MRI sessions and the breaks was calculated for both groups. The statistical analysis shows a strong correlation between the number of MRI sessions and the apparition of cells with few (one or two) breaks. However, there was no correlation between the number of MRI sessions and the number of cells with three or more chromosome breaks. *MRI*, Magnetic resonance imaging

The total number of damaged cells increased by 3.2% (95% CI 1.5–4.8%) per MRI (Fig. 2d–h) (*p* < 0.001); this increase being higher during the first ten MRI sessions than during the last ten ones (*p* for interaction = 0.016). This result suggests an “early effect” of repetitive MRI exposure with a limited accumulation of damaged cells. The increase in damaged cells was related to the MRI exams received during the previous 6 months (increase 6.9%, 95% CI 1.2–12.6%, per MRI) rather than to the ones received earlier (increase 1.3%, 95 CI 0.0–2.6%, per MRI), suggesting a transient and mid-term effect of exposure. There was a significant association between the number of cells with a low amount of damage (one or two breaks) and repeated MRI sessions, whereas there was no significant correlation between the frequency of cells with more extensive damage (more than three breaks) and the number of MRI sessions completed (Fig. 3).

#### The chromosomal changes reach a plateau after 20 MRI sessions

**Fig. 4:**
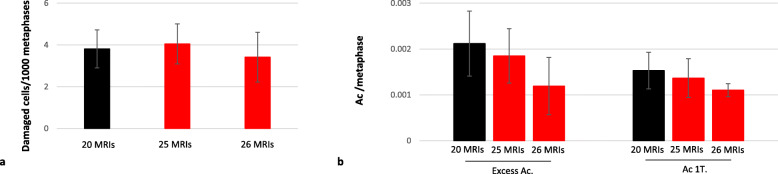
Stabilisation of cytogenetic parameters after 20 MRI sessions. Further analysis was conducted after six supplementary MRI sessions. Evolution of damaged cells (**a**) and type E acentric fragments/1-T acentric fragments (**b**) between 20 and 25–26 MRI sessions. All parameters remained stable. *Ac*, Acentric fragment; *MRI*, Magnetic resonance imaging; *T*, Telomere

We show that 20 repetitive MRI sessions, over 2 to 3 years, were associated with the doubling of the frequency of damaged cells. The period of 2 to 3 years is a very wide window for the detection of chromosome aberrations as adjustments are necessary after a period of 1 year to account for lymphocyte regeneration after a single exposure. As such, the evolution of the cytogenetic parameters was verified after supplementary MRI sessions (Fig. 4). The frequency of damaged cells remained stable between 20 and 25–26 MRI sessions (Fig. 4a), as well as the frequency of various acentric fragments (Fig. 4b). After 20 MRI sessions, the CA frequency reached a plateau and additional exposure with the same timing did not show further changes.

### CT scans induced much stronger chromosomal destabilisation and chromosome fusions than MRI

**Fig. 5:**
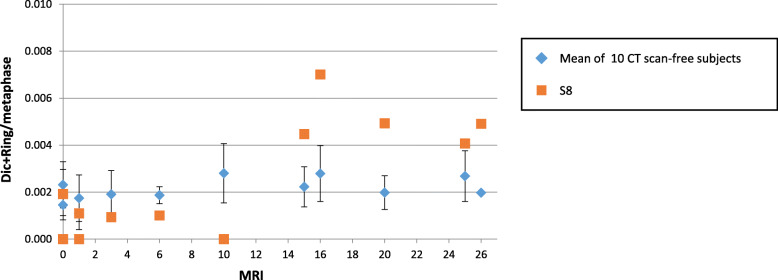
Increase in the frequency of dicentric chromosomes after CT. The frequency of dicentric and ring chromosomes is presented up until 26 MRI sessions for the ten MRI-only volunteers and for the subject number 8, a volunteer exposed to computed tomography examinations (8 between the 10 and 25 MRI sessions) for an unexpected diagnostic assessment of a non-brain pathology during the originally planned MRI sessions schedule. *Dic*, Dicentric chromosomes; *MRI*, Magnetic resonance imaging

Volunteer S8 underwent five whole body CT scans (ionising radiation exposure) between the 10th and 15th MRI. The first CA analysis after the start of the scan sessions showed a jump in the frequency of Dics (Fig. 5), as well as other parameters (frequency of acentric fragments/damaged cells/total DNA breaks) (data not shown), between MRI sessions 10 and 15 interspersed with the five CT scans over less than 1 year. Interestingly, the abrupt increase of CAs due to the CT sessions was much higher than the increase due to the previous ten MRI sessions and, in fact, higher than that following 20 MRI sessions.

## Discussion

This study is the first to perform a follow-up of the chromosomal integrity of volunteers exposed to repetitive 3-T MRI using the detection of CAs. While we do not report any change after a single MRI session, repeated exposure was associated with an increase in the frequency of chromosomal deletions. The study of Fatahi et al. [5] is the only other one to have studied multi-MRI exposure, as they quantified DSBs in subjects previously exposed to repetitive sessions of 7-T MRI. Their results clearly showed no difference in γ-H2AX or micronuclei frequency between the controls and the MRI-exposed group. However, there was no data concerning the exposed group prior to exposure. As such, the heterogeneity of the number of exposures and their duration within the group, as well as the low sensitivity of the micronuclei assay could explain the absence of a significant difference.

An aging effect could be evoked as 2 to 3 years have passed during the course of our MRI study [18]. Indeed, the level of evidence provided by a longitudinal study of healthy volunteers at each exposure is very high. It is higher than that of a study comparing one or more exposed groups to a control group at a single time point. In the literature, the longitudinal follow-up of CAs in similar but non-exposed subjects has not been reported and the exact correlation between aging and the frequency of unstable CAs over such a short time period (2 to 3 years) is not defined.

Many genetic and environmental factors do contribute to the increase of CAs with age, which could not all be controlled in this study. Indeed, in absolute terms, the longitudinal study of a non-MRI exposed control group would help estimate more precisely the magnitude of the MRI-related changes. A period of 3 to 5 years is a very short time period for an individual whose life expectancy is 80 years. As such, data from the literature confirms that it is unrealistic to expect observed age-related changes in this age group over such a short time period. In the most complete aging study conducted on a large population using the micronuclei technique, Fenech et al. [19] reported an increase in micronuclei frequency between 30 and 40 years of age of 1.06% for men and 1.23% for women, whereas Ganguly et al. [20] reported an increase in all types of damaged cells with age, with an increase of 7.3% per year. In our study, the increase in the frequency of cells containing at least one break from the initial value was equal to a factor of 2.07 after 20 MRI sessions, corroborating that the frequency of damaged cells doubled after 20 MRI sessions over a period of 2 to 3 years. This factor is much higher than the age-dependent factor, thus suggesting a genotoxic effect of MRI. Another study [21] predicted an increase from 0.1162 to 0.1456 total acentric fragments for 100 cells between the ages of 30 and 40 years. In our study, type E acentric fragments increased by a factor of 2.9 after 20 MRI sessions, equivalent to their tripling, very low in comparison with CT scans.

Moreover, and interestingly, the profile of CA evolution in MRI-exposed volunteers was not the same as those usually observed after CT scans [22–27], as we mainly detected chromosomal deletions and no increase in Dics, the hallmark of irradiation. We hypothesise that the number of DSBs induced per cell by repeated MRIs is too low to induce chromosomal fusion. Alternatively, biodosimetry studies are generally performed above 100 mGy, and lower-dose studies are lacking. Very low dose (*i.e.*, a few mGy) could trigger a very low number of DSBs, with a very low probability of having two misrepaired DSBs in the same cell. Indeed, it appears that most DSBs are immediately repaired after irradiation, leading either to recombination or restoration of chromosomal integrity, the latter being undetectable [28]. Complementary data concerning the induction of acentric fragments after very low-dose exposure to ionising radiation would lend support to this hypothesis and might be a prerequisite to perform a “dose-equivalent” estimation of MRI effects.

The frequency of damaged cells, as well as that of acentric fragments, appears to reach a plateau after 20 MRI sessions. In regard to lymphocyte turnover, these observations suggest that acentric fragments are better transmitted to daughter cells than Dics. Most studies in the literature have compared the transmission of Dics, that decrease by 50% at each cell division, *versus* translocations that remain stable if there are no Dics or rings in the same cell [17, 29, 30]. Acentric fragments, although not as stable as those chromosomes that have been translocated, appear to be more stable and transmissible than Dics and may last for a few cell divisions. Indeed, Al-Achkar et al. [31] reported a detectable decrease in total acentric fragments after a minimum of three cell divisions. These results are coherent with our observations of an increase in the number of damaged cells when 20 MRI sessions were completed, that is, 2 to 3 years after the first exposure. This time period corresponds to a sufficient number of divisions of the lymphocytes to lose the acentric fragments, which is necessary to observe a plateau in the frequency of acentric fragments.

Further analysis is needed to validate the “semi-stable” property of acentric fragments while increasing the number of events analysed. The cytokinesis-blocked micronucleus assay is frequently used with cytochalasin blockage after 44 h (one or two cell divisions) or 68 h (one, two, or three cell divisions) of cultivation depending on weekly logistic constraints. Most micronuclei contain acentric fragments. The relatively good stability of acentric fragments during cell division and inter-individual variation could partially explain the contradictory results published to date. The cytokinesis-blocked micronucleus assay upgraded with telomere and centromere staining might be a good alternative for large screening and acentric-fragment transmissibility investigations.

Various hypotheses need to be explored to explain the exact cause of the increase of CAs following MRI, and could include the perturbation of the DNA repair machinery, alteration of the mitotic processes, or the accumulation of oxidative stress. Additionally, better control of the multiple factors involved in the inter- and intra-individual variability of CAs would assist in estimating more precisely the magnitude of MRI-related effects.

In summary, after one session, 3-T MRI (which corresponds to standard medical use) is a very safe imaging technique with undetectable changes at the level of the chromosome. Repetitive exposure (20 MRIs over 2–3 years) leads to an increase in the frequency of damaged cells with one DSB that remains very small in comparison with CT scans. Efforts will now focus on validating the transient increase in acentric fragments (2–3 years) by continuing to follow the volunteers until the last exposure (50 MRIs).

## Supplementary Information

**Additional file 1: Figure S1.** Technique of fluorescence *in situ* hybridisation (FISH) of telomeres and centromeres on metaphases obtained after the dicentric assay (DCA). The DCA was performed on blood samples at various times before or after MRI. Then, FISH staining of telomeres and centromeres was carried out to detect chromosomal aberrations on a total of 143,872 metaphases up to 20 MRI sessions; 9 multi-aberrant cells (Rogue cells) were excluded. **a.** FISH staining of telomeres and centromeres allows the visualisation of centromeres in green and telomeres in red on each chromosome, driving the scoring of DNA DSBs. **b.** An example of stained metaphases is shown with 46 chromosomes. **c.** A metaphase with multiple CAs is shown. Dicentric (Dic) and tricentric (Tric) chromosomes are indicated, as well as rings. **d.** The table presents the various unstable aberrations detectable by FISH staining. The corresponding number of DNA DSBs for each aberration is also indicated. **Figure S2.** Planning of the MRI sessions and blood sampling for the 13 subjects. Eleven of the 13 subjects were exposed to 25 MRI exams over three to four years. Three samplings and cytogenetics analysis were performed before any MRI exposure (0 MRI) to check background heterogeneity. Blood sampling was performed the day of the following MRI, just before the planned exam, to examine the mid-term effects of repetitive MRI exposure, except after the 1^st^ and the 16^th^ MRI sessions, for which sampling was performed just after exposure. **Figure S3.** No accumulation of transmissible chromosome rearrangements after chromosome painting. Chromosome painting of chromosomes 1, 4, and 11 was performed on S7 and S8 before MRI and after 16 MRI sessions. The genome fraction painted is equivalent to 0.1921 of the total genome. **a.** The same metaphase is stained using the FISH method for telomeres and centromeres and by the chromosome painting technique. **b.** The scoring of DNA DSBs is shown in the table, as well as the total metaphases scored. **Supplementary table 1 a :** Summary of abnormal metaphasis and terminal deletions donors S1-S2-S3. **Supplementary table 1 b :** Summary of abnormal metaphasis and terminal deletions donors S4-S5-S6. **Supplementary table 1 c :** Summary of abnormal metaphasis and terminal deletions donors S7-S8-S9. **Supplementary table 1 d :** Summary of abnormal metaphasis and terminal deletions donors S10-S11-S12-S13

## Data Availability

Most data generated or analysed during this study are included in this published article (and its supplementary information files). Additional datasets used and/or analysed during the current study are available from the corresponding author on reasonable request.

## Abbreviations

3D: Three-dimensional
CAs: Chromosomal aberrations
CI: Confidence interval
CT: Computed tomography
Dic: Dicentric (fused chromosomes with two centromeres)
DNA: Deoxyribonucleic acid
DSB: Double-strand DNA break
FISH: Fluorescence *in situ* hybridisation
FITC: Fluorescein isothiocyanate
MRI: Magnetic resonance imaging
PNA: Peptide nucleic acid
SSB: Single-strand DNA break
TC: Telomere/centromere
Tric: Tricentric (fused chromosomes with three centromeres)
