# Supplementary material for: The effects of repeated brain MRI on chromosomal damage
Source: Eur Radiol Exp. 2022 Mar 3;6:12. doi: 10.1186/s41747-022-00264-2 (PMC8891399; doi:10.1186/s41747-022-00264-2)
Supplement: Supplementary file 1 — Additional file 1: Figure S1. Technique of fluorescence in situ hybridisation (FISH) of telomeres and centromeres on metaphases obtained after the dicentric assay (DCA). The DCA was performed on blood samples at various times before or after MRI. Then, FISH staining of telomeres and centromeres was carried out to detect chromosomal aberrations on a total of 143,872 metaphases up to 20 MRI sessions; 9 multi-aberrant cells (Rogue cells) were excluded. a. FISH staining of telomeres and centromeres allows the visualisation of centromeres in green and telomeres in red on each chromosome, driving the scoring of DNA DSBs. b. An example of stained metaphases is shown with 46 chromosomes. c. A metaphase with multiple CAs is shown. Dicentric (Dic) and tricentric (Tric) chromosomes are indicated, as well as rings. d. The table presents the various unstable aberrations detectable by FISH staining. The corresponding number of DNA DSBs for each aberration is also indicated. Figure S2. Planning of the MRI sessions and blood sampling for the 13 subjects. Eleven of the 13 subjects were exposed to 25 MRI exams over three to four years. Three samplings and cytogenetics analysis were performed before any MRI exposure (0 MRI) to check background heterogeneity. Blood sampling was performed the day of the following MRI, just before the planned exam, to examine the mid-term effects of repetitive MRI exposure, except after the 1st and the 16th MRI sessions, for which sampling was performed just after exposure. Figure S3. No accumulation of transmissible chromosome rearrangements after chromosome painting. Chromosome painting of chromosomes 1, 4, and 11 was performed on S7 and S8 before MRI and after 16 MRI sessions. The genome fraction painted is equivalent to 0.1921 of the total genome. a. The same metaphase is stained using the FISH method for telomeres and centromeres and by the chromosome painting technique. b. The scoring of DNA DSBs is shown in the table, as well as the total metaphas [file 41747_2022_264_MOESM1_ESM.docx]

**ELECTRONIC SUPPLEMENTARY MATERIAL**


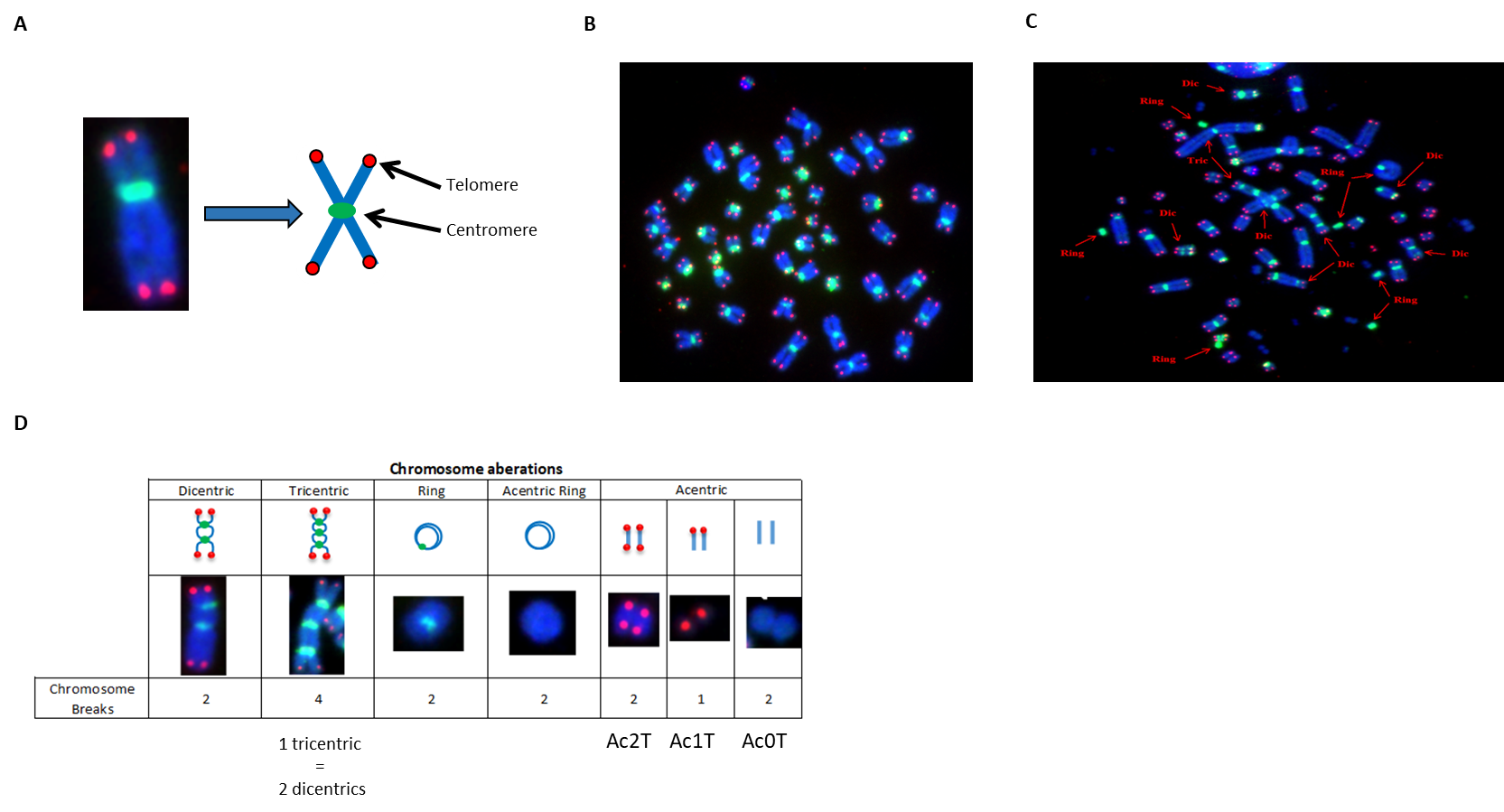


**Figure S1. Technique of fluorescence *in situ* hybridization (FISH) of telomeres and centromeres on metaphases obtained after the DCA.** The DCA was performed on blood samples at various times before or after MRI. Then, FISH staining of telomeres and centromeres was carried out to detect chromosomal aberrations on a total of 143,872 metaphases up to 20 MRI sessions; 9 multi-aberrant cells (Rogue cells) were excluded. **A.** FISH staining of telomeres and centromeres allows the visualisation of centromeres in green and telomeres in red on each chromosome, driving the scoring of DNA DSBs. **B.** An example of stained metaphases is shown with 46 chromosomes. **C.** A metaphase with multiple CAs is shown. Dicentric (Dic) and tricentric (Tric) chromosomes are indicated, as well as rings. **D.** The table presents the various unstable aberrations detectable by FISH staining. The corresponding number of DNA DSBs for each aberration is also indicated.


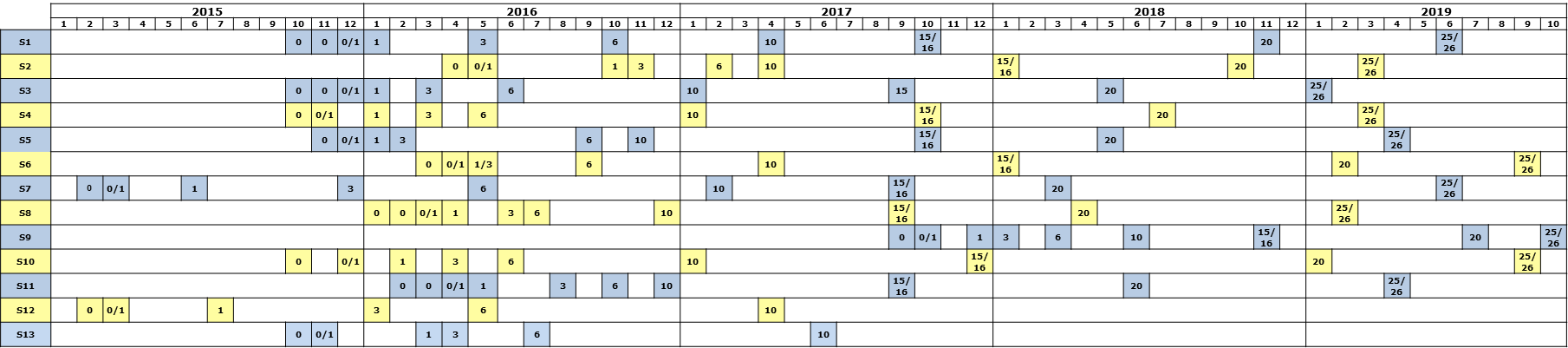


**Figure S2. Planning of the MRI sessions and blood sampling for the 13 subjects.** Eleven of the 13 subjects were exposed to 25 MRI exams over three to four years. Three samplings and cytogenetics analysis were performed before any MRI exposure (0 MRI) to check background heterogeneity. Blood sampling was performed the day of the following MRI, just before the planned exam, to examine the mid-term effects of repetitive MRI exposure, except after the 1^st^ and the 16^th^ MRI sessions, for which sampling was performed just after exposure.


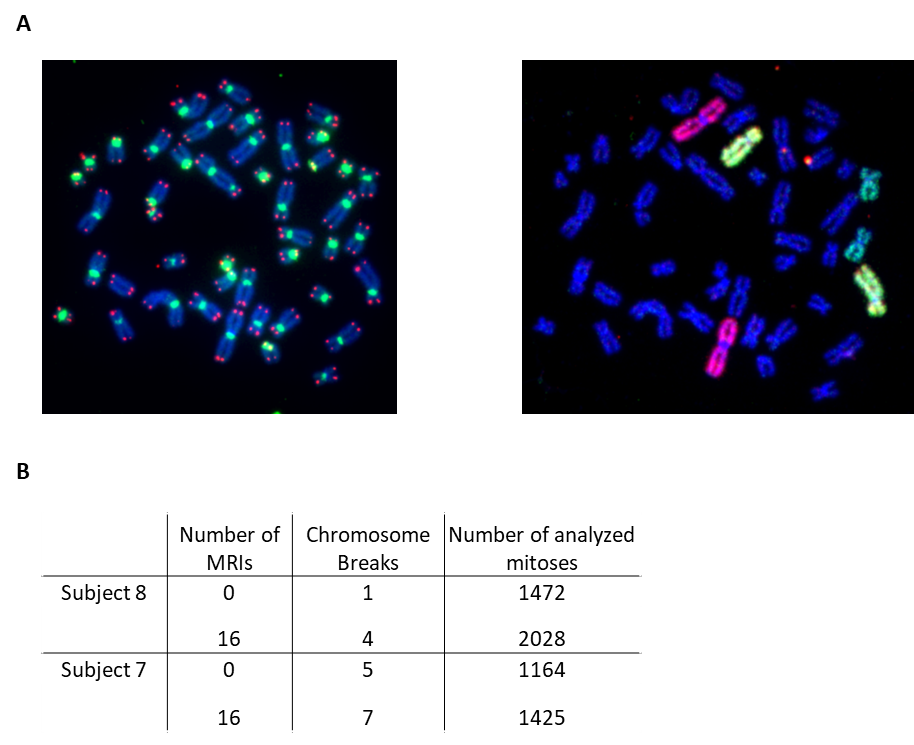


**Figure S3. No accumulation of transmissible chromosome rearrangements after chromosome painting.** Chromosome painting of chromosomes 1, 4, and 11 was performed on S7 and S8 before MRI and after 16 MRI sessions. The genome fraction painted is equivalent to 0.1921 of the total genome. **A.** The same metaphase is stained using the FISH method for telomeres and centromeres and by the chromosome painting technique. **B.** The scoring of DNA DSBs is shown in the table, as well as the total metaphases scored.

| **Donors** | **Sessions** | **Number of MRIs** | **Analysed metaphasis** | **Damaged cells** | **Acentric 1T : terminal deletion** |
| --- | --- | --- | --- | --- | --- |
| **S1** | V-30 | 0 MRI | 1174 | 4 | 0 |
|  | V-15 | 0 MRI | 1007 | 1 | 0 |
|  | V0 pre-MRI | 0 MRI | 846 | 1 | 0 |
|  | V0 post-MRI | 1 MRI | 987 | 0 | 0 |
|  | V1 | 1 MRI | 988 | 0 | 0 |
|  | V3 | 3 MRIs | 1019 | 1 | 0 |
|  | V6 | 6 MRIs | 1050 | 2 | 0 |
|  | V10 | 10 MRIs | 1021 | 0 | 0 |
|  | V15 pre-MRI | 15 MRIs | 1022 | 2 | 1 |
|  | V15 post-MRI | 16 MRIs | 955 | 7 | 2 |
|  | V20 | 20 MRIs | 1043 | 3 | 2 |
|  | V25 pre-MRI | 25 MRIs | 1042 | 3 | 1 |
|  | V25 post-MRI | 26 MRIs | 1017 | 1 | 0 |
| **S2** | V-30 | 0 MRI | 933 | 2 | 0 |
|  | V-15 | 0 MRI | 1082 | 1 | 0 |
|  | V0 pre-MRI | 0 MRI | 1059 | 0 | 0 |
|  | V0 post-MRI | 1 MRI | 952 | 0 | 0 |
|  | V1 | 1 MRI | 1090 | 0 | 0 |
|  | V3 | 3 MRIs | 901 | 2 | 0 |
|  | V6 | 6 MRIs | 1031 | 1 | 0 |
|  | V10 | 10 MRIs | 930 | 3 | 1 |
|  | V15 pre-MRI | 15 MRIs | 992 | 3 | 1 |
|  | V15 post-MRI | 16 MRIs | 1049 | 5 | 0 |
|  | V20 | 20 MRIs | 1144 | 5 | 2 |
|  | V25 pre-MRI | 25 MRIs | 1020 | 6 | 3 |
|  | V25 post-MRI | 26 MRIs | 1029 | 2 | 2 |
| **S3** | V-30 | 0 MRI | 1148 | 4 | 0 |
|  | V-15 | 0 MRI | 1005 | 1 | 0 |
|  | V0 pre-MRI | 0 MRI | 2038 | 6 | 0 |
|  | V0 post-MRI | 1 MRI | 1027 | 6 | 0 |
|  | V1 | 1 MRI | 2118 | 6 | 0 |
|  | V3 | 3 MRIs | 1013 | 2 | 1 |
|  | V6 | 6 MRIs | 1008 | 5 | 1 |
|  | V10 | 10 MRIs | 1032 | 7 | 1 |
|  | V15 pre-MRI | 15 MRIs | 1110 | 4 | 0 |
|  | V15 post-MRI | 16 MRIs | 1041 | 2 | 0 |
|  | V20 | 20 MRIs | 1025 | 8 | 3 |
|  | V25 pre-MRI | 25 MRIs | 1028 | 4 | 2 |
|  | V25 post-MRI | 26 MRIs | 1121 | 7 | 2 |

Supplementary table 1 a : Summary of abnormal metaphasis and terminal deletions donors S1-S2-S3

| **Donors** | **Sessions** | **Number of MRIs** | **Analysed metaphasis** | **Damaged cells** | **Acentric 1T : terminal deletion** |
| --- | --- | --- | --- | --- | --- |
| **S4** | V-30 | 0 MRI | 901 | 0 | 0 |
|  | V-15 | 0 MRI | 942 | 3 | 0 |
|  | V0 pre-MRI | 0 MRI | 996 | 2 | 1 |
|  | V0 post-MRI | 1 MRI | 1011 | 0 | 0 |
|  | V1 | 1 MRI | 1056 | 4 | 1 |
|  | V3 | 3 MRIs | 1016 | 4 | 2 |
|  | V6 | 6 MRIs | 1022 | 3 | 0 |
|  | V10 | 10 MRIs | 1017 | 2 | 0 |
|  | V15 pre-MRI | 15 MRIs | 1041 | 2 | 1 |
|  | V15 post-MRI | 16 MRIs | 1059 | 2 | 0 |
|  | V20 | 20 MRIs | 1091 | 6 | 1 |
|  | V25 pre-MRI | 25 MRIs | 1029 | 4 | 1 |
|  | V25 post-MRI | 26 MRIs | 1039 | 0 | 0 |
| **S5** | V-30 | 0 MRI | 1373 | 6 | 0 |
|  | V-15 | 0 MRI | 1039 | 1 | 0 |
|  | V0 pre-MRI | 0 MRI | 1000 | 2 | 1 |
|  | V0 post-MRI | 1 MRI | 1054 | 3 | 0 |
|  | V1 | 1 MRI | 887 | 1 | 0 |
|  | V3 | 3 MRIs | 1043 | 0 | 0 |
|  | V6 | 6 MRIs | 1016 | 4 | 3 |
|  | V10 | 10 MRIs | 1046 | 2 | 0 |
|  | V15 pre-MRI | 15 MRIs | 1056 | 5 | 1 |
|  | V15 post-MRI | 16 MRIs | 924 | 6 | 3 |
|  | V20 | 20 MRIs | 1005 | 4 | 2 |
|  | V25 pre-MRI | 25 MRIs | 1019 | 7 | 2 |
|  | V25 post-MRI | 26 MRIs | 1046 | 2 | 0 |
| **S6** | V-30 | 0 MRI | 1028 | 1 | 0 |
|  | V-15 | 0 MRI | 1038 | 2 | 0 |
|  | V0 pre-MRI | 0 MRI | 1056 | 3 | 0 |
|  | V0 post-MRI | 1 MRI | 1038 | 0 | 0 |
|  | V1 | 1 MRI | 1016 | 1 | 1 |
|  | V3 | 3 MRIs | 1059 | 1 | 1 |
|  | V6 | 6 MRIs | 1038 | 5 | 3 |
|  | V10 | 10 MRIs | 1008 | 0 | 0 |
|  | V15 pre-MRI | 15 MRIs | 994 | 3 | 0 |
|  | V15 post-MRI | 16 MRIs | 1025 | 5 | 2 |
|  | V20 | 20 MRIs | 1011 | 1 | 1 |
|  | V25 pre-MRI | 25 MRIs | 1035 | 6 | 2 |
|  | V25 post-MRI | 26 MRIs | 1002 | 3 | 1 |

Supplementary table 1 b : Summary of abnormal metaphasis and terminal deletions donors S4-S5-S6

| **Donors** | **Sessions** | **Number of MRIs** | **Analysed metaphasis** | **Damaged cells** | **Acentric 1T : terminal deletion** |
| --- | --- | --- | --- | --- | --- |
| **S7** | V-30 | 0 MRI | 1482 | 3 | 1 |
|  | V-15 | 0 MRI | 2114 | 12 | 0 |
|  | V0 pre-MRI | 0 MRI | 2040 | 1 | 0 |
|  | V0 post-MRI | 1 MRI | 1068 | 2 | 0 |
|  | V1 | 1 MRI | 874 | 2 | 0 |
|  | V3 | 3 MRIs | 1003 | 3 | 0 |
|  | V6 | 6 MRIs | 1105 | 6 | 3 |
|  | V10 | 10 MRIs | 1087 | 8 | 0 |
|  | V15 pre-MRI | 15 MRIs | 1022 | 8 | 1 |
|  | V15 post-MRI | 16 MRIs | 1144 | 9 | 2 |
|  | V20 | 20 MRIs | 1125 | 3 | 1 |
|  | V25 pre-MRI | 25 MRIs | 1002 | 1 | 0 |
|  | V25 post-MRI | 26 MRIs | 1027 | 4 | 2 |
| **S8** | V-30 | 0 MRI | 1042 | 2 | 0 |
|  | V-15 | 0 MRI | 1034 | 1 | 1 |
|  | V0 pre-MRI | 0 MRI | 1019 | 0 | 0 |
|  | V0 post-MRI | 1 MRI | 914 | 1 | 0 |
|  | V1 | 1 MRI | 1020 | 1 | 0 |
|  | V3 | 3 MRIs | 1071 | 2 | 1 |
|  | V6 | 6 MRIs | 995 | 3 | 2 |
|  | V10 | 10 MRIs | 1001 | 0 | 0 |
|  | V15 pre-MRI | 15 MRIs | 3357 | 29 | 9 |
|  | V15 post-MRI | 16 MRIs | 2996 | 32 | 4 |
|  | V20 | 20 MRIs | 1014 | 5 | 1 |
|  | V25 pre-MRI | 25 MRIs | 1229 | 8 | 3 |
|  | V25 post-MRI | 26 MRIs | 1019 | 8 | 1 |
| **S9** | V-30 | 0 MRI | 1081 | 2 | 0 |
|  | V-15 | 0 MRI | 912 | 1 | 0 |
|  | V0 pre-MRI | 0 MRI | 998 | 2 | 1 |
|  | V0 post-MRI | 1 MRI | 970 | 2 | 0 |
|  | V1 | 1 MRI | 1077 | 1 | 1 |
|  | V3 | 3 MRIs | 1055 | 3 | 0 |
|  | V6 | 6 MRIs | 1027 | 2 | 0 |
|  | V10 | 10 MRIs | 1142 | 6 | 2 |
|  | V15 pre-MRI | 15 MRIs | 1034 | 3 | 1 |
|  | V15 post-MRI | 16 MRIs | 1095 | 2 | 1 |
|  | V20 | 20 MRIs | 1059 | 3 | 2 |
|  | V25 pre-MRI | 25 MRIs | 1034 | 2 | 1 |
|  | V25 post-MRI | 26 MRIs | 585 | 3 | 2 |

Supplementary table 1 c : Summary of abnormal metaphasis and terminal deletions donors S7-S8-S9

| **Donors** | **Sessions** | **Number of MRIs** | **Analysed metaphasis** | **Damaged cells** | **Acentric 1T : terminal deletion** |
| --- | --- | --- | --- | --- | --- |
| **S10** | V-30 | 0 MRI | 1305 | 3 | 0 |
|  | V-15 | 0 MRI | 891 | 1 | 0 |
|  | V0 pre-MRI | 0 MRI | 1047 | 5 | 0 |
|  | V0 post-MRI | 1 MRI | 1043 | 4 | 0 |
|  | V1 | 1 MRI | 1015 | 2 | 1 |
|  | V3 | 3 MRIs | 1010 | 2 | 0 |
|  | V6 | 6 MRIs | 1264 | 5 | 0 |
|  | V10 | 10 MRIs | 972 | 5 | 0 |
|  | V15 pre-MRI | 15 MRIs | 1028 | 1 | 0 |
|  | V15 post-MRI | 16 MRIs | 1027 | 0 | 0 |
|  | V20 | 20 MRIs | 1001 | 3 | 1 |
|  | V25 pre-MRI | 25 MRIs | 1147 | 3 | 1 |
|  | V25 post-MRI | 26 MRIs | 1067 | 5 | 1 |
| **S11** | V-30 | 0 MRI | 1020 | 1 | 0 |
|  | V-15 | 0 MRI | 1011 | 0 | 0 |
|  | V0 pre-MRI | 0 MRI | 1074 | 2 | 0 |
|  | V0 post-MRI | 1 MRI | 1022 | 1 | 0 |
|  | V1 | 1 MRI | 1034 | 0 | 0 |
|  | V3 | 3 MRIs | 1084 | 0 | 0 |
|  | V6 | 6 MRIs | 1082 | 5 | 1 |
|  | V10 | 10 MRIs | 1015 | 3 | 0 |
|  | V15 pre-MRI | 15 MRIs | 1021 | 2 | 0 |
|  | V15 post-MRI | 16 MRIs | 1021 | 3 | 1 |
|  | V20 | 20 MRIs | 1046 | 3 | 1 |
|  | V25 pre-MRI | 25 MRIs | 1002 | 3 | 1 |
|  | V25 post-MRI | 26 MRIs | 1044 | 2 | 0 |
| **S12** | V-30 | 0 MRI | 838 | 1 | 0 |
|  | V-15 | 0 MRI | 1128 | 3 | 1 |
|  | V0 pre-MRI | 0 MRI | 926 | 1 | 0 |
|  | V0 post-MRI | 1 MRI | 1062 | 2 | 0 |
|  | V1 | 1 MRI | 874 | 1 | 0 |
| **S13** | V-30 | 0 MRI | 855 | 4 | 0 |
|  | V-15 | 0 MRI | 1017 | 1 | 0 |
|  | V0 pre-MRI | 0 MRI | 1050 | 1 | 0 |
|  | V0 post-MRI | 1 MRI | 1023 | 3 | 0 |
|  | V1 | 1 MRI | 1043 | 3 | 0 |

Supplementary table 1 d : Summary of abnormal metaphasis and terminal deletions donors S10-S11-S12-S13
